# Germline progenitors and oocyte production in the honeybee queen ovary

**DOI:** 10.1093/genetics/iyad138

**Published:** 2023-07-24

**Authors:** Georgia Cullen, Joshua B Gilligan, Joseph G Guhlin, Peter K Dearden

**Affiliations:** Laboratory for Evolution and Development, Biochemistry Department, University of Otago, Dunedin, 9054, Aotearoa-New Zealand; Laboratory for Evolution and Development, Biochemistry Department, University of Otago, Dunedin, 9054, Aotearoa-New Zealand; Biological Heritage National Science Challenge, Biochemistry Department, University of Otago, Dunedin, 9054, Aotearoa-New Zealand; Laboratory for Evolution and Development, Biochemistry Department, University of Otago, Dunedin, 9054, Aotearoa-New Zealand; Genomics Aotearoa, Biochemistry Department, University of Otago, Dunedin, 9054, Aotearoa-New Zealand; Laboratory for Evolution and Development, Biochemistry Department, University of Otago, Dunedin, 9054, Aotearoa-New Zealand; Biological Heritage National Science Challenge, Biochemistry Department, University of Otago, Dunedin, 9054, Aotearoa-New Zealand; Genomics Aotearoa, Biochemistry Department, University of Otago, Dunedin, 9054, Aotearoa-New Zealand

**Keywords:** honeybees, ovary, cell division, reproductive rate, gene expression

## Abstract

Understanding the reproduction of honeybee queens is crucial to support populations of this economically important insect. Here we examine the structure of the honeybee ovary to determine the nature of the germline progenitors in the ovary. Using a panel of marker genes that mark somatic or germline tissue in other insects we determine which cells in the honeybee ovary are somatic and which germline. We examine patterns of cell division and demonstrate that, unlike *Drosophila*, there is no evidence of single germline stem cells that provide the germline in honeybees. Germline progenitors are clustered in groups of 8 cells, joined by a polyfusome, and collections of these, in each ovariole, appear to maintain the germline during reproduction. We also show that these 8-cell clusters can divide and that their division occurs such that the numbers of germline progenitors are relatively constant over the reproductive life of queen honeybees. This information helps us to understand the diversity of structures in insect reproduction, and provide information to better support honeybee reproduction.

## Teaser

Cullen *et al*. examine the structure of the honeybee ovary to determine the nature of ovarian germline progenitors, finding that they exist as clusters of eight cells joined by a cytoplasmic bridge. These cell clusters can divide, and their division is enough to maintain the reproductive output of honeybee queens over their life.

## Introduction

Honeybees are economically important insects, providing hive products such as honey, wax, and propolis, as well as pollination services estimated at US$34 billion/year in 2012 (Jordan *et al*. 2021). Because of their eusocial life history strategy (Tautz 2008), honeybee reproduction depends, to a large extent, on a single queen bee present in each hive. Reproduction, and thus productivity of a bee colony depends on the activity of the queen ovary. Given recent challenges to both managed and unmanaged honeybee populations (Potts *et al*. 2010), such as varroa mite vectored disease and the widespread use of insecticides, it is important to know how best to support honeybee queen reproduction.

Despite the importance of honeybees, little is known about the structure and function of honeybee ovaries. We do not understand the control of stem cell division in the ovary, or even where the germline stem cells are placed. Knowing more about the structure and function of the queen ovary may help us determine the best time to intervene in honeybee queen development to maximize the rate of reproduction, quality of offspring, and total reproductive output.

Insect ovaries are categorized into structural groups (Büning 1994). Honeybees have polytrophic meroistic ovaries (Gutzeit *et al*. 1993), meaning the oocyte is nourished by a set of nurse cells (trophocytes) (Büning 1994). These are attached to the oocyte via cytoplasmic bridges (polyfusomes and ring canals (Telfer 1975)) that travel with each oocyte down the ovariole before dying just before egg laying.

Honeybees are holometabolous insects and their polytrophic meroistic ovary structure has meant they are often compared to *Drosophila melanogaster* (Kirilly and Xie 2007). It is clear that even amongst this ovary type and holometabolous insects themselves, the structure and function of the ovary differ.

These differences are no surprise given the different life-history contexts these species exist in; honeybee queens, in particular, are reputed to be able to lay between 500 and 3000 (Allen 1955; Harbo 1986; Avni *et al*. 2014) eggs a day and to live 1–5 years (Sammataro and Avitabile 1998; Page Jr and Peng 2001) while wild-caught *D. melanogaster*, in a laboratory environment, lay between 1,000–2,000 eggs over their approx. 60–70-day life (Klepsatel *et al*. 2013).

In honeybees, part of this reproductive output is explained by the number of ovarioles each queen possesses. Ovarioles are the functional units of the ovary, each being a long strand of cells. At the anterior end is the terminal filament, and then, toward the posterior, the germarium, followed by the vitellarium, from which mature eggs are released. Experiments to determine the range and number of ovarioles in honeybee ovaries indicate they contain between 233 and 438 (median 320) in each of 2 ovaries (Jackson *et al*. 2011), whereas in *Drosophila melanogaster*, the number of ovarioles varies between 18 and 20, in *Tribolium castaneum* (red flour beetle) it is 8, in *Bombyx mori* (silk moth) it is 8, and in non-Apis Hymenoptera, numbers range from 2 to 10,000 ovarioles. Queen ants and bees make up the larger numbers of ovarioles, but most species have between 8 and 100 (Church *et al*. 2021).

In *D. melanogaster*, the germarium in each ovariole contains 2–3 germline stem cells whose division is crucial to reproductive output (reviewed in McLaughlin and Bratu (2015). These cells divide with 1 daughter cell remaining a stem cell, and the other forming the first stage of oocyte production, a cystoblast. Cystoblasts divide 4 times, producing 16 cystocytes that remain cytoplasmically connected by cytoplasmic bridges, named ring canals, which are organized by a structure known as a fusome. One of the cystocytes, determined through a complex process, develops into the oocyte, the others form nurse cells or trophoblasts. As the oocyte develops, these nurse cells divide and endoreplicate their genomes, becoming polyploid, and generate proteins and RNA which are transported into the oocyte, both providing nutrition and patterning the oocyte. As the oocyte matures and is readied to be laid, the nurse cells contract, dumping their cytoplasm into the oocyte, and dying; this provisioned and patterned egg is then laid.

While this process has been well studied in *Drosophila*, the ovaries of *Apis* appear to have significant differences from the *Drosophila* model. Previous studies in honeybees have suggested that germline cells are present in the terminal filament of the ovary (Gutzeit *et al*. 1993; Tanaka and Hartfelder 2004), as has been described for another bee species; Osmia (Van Eeckhoven and Duncan 2020). Histochemical and contrast studies indicate that the germarium of honeybees is filled with resting germline cells, but it is not clear how these cells are arranged and whether they divide (Büning 1994). At the boundary of the germarium and the vitellarium, it is reported that the cells are arranged into rosettes, joined by a polyfusome, prefiguring the development of a nurse/oocyte cluster (Hegner 1915; Tanaka and Hartfelder 2004). The cysts in *Apis* also form differently from *D. melanogaster*, with each nurse cell cluster separated by a narrow neck from the oocyte (Tanaka and Hartfelder 2004; Dearden 2006; Aamidor *et al*. 2022).

To begin to understand the placement of germline and somatic cells in the honeybee ovary, and the source of its remarkable fecundity, we examined the structure of honeybee ovarioles at high resolution. We also classified cell types in the ovary as somatic or germline and examined the placement and timing of cell divisions in the ovary as a way to understand reproduction in honeybees.

## Materials and methods

### Honeybees

Actively laying *Apis mellifera* queens were selected from different hives across Dunedin, Otago. Replicates of each experiment were carried out with queens that were not closely related. For immunohistochemistry experiments, tissues came from 5 replicate queens. 5-ethynyl-2’-deoxyuridine (EdU) experiments used 5 replicates for 24-hour experiments and 3 for 48-hour experiments. *In situ* hybridization involved 18 replicate queens, at least 3 per gene. Multiple ovarioles for each queen were examined in each case. For polyfusome counting experiments, 6 queens were divided into 2 groups (3 per group), based on their age. Old queens are defined as being in their second season, having over-wintered and being older than 1 year, and young queens as in their first season, 3–10 months old. Multiple ovarioles from each queen were examined.

### Dissection and fixation

Laying adult queen honeybees were dissected as soon as possible after hive removal. Unlike previous experiments (Aamidor *et al*. 2022), and due to concerns about tissue damage, neither bees nor ovaries were ever subjected to freezing unless fixed and stored in methanol. Queens were anesthetized by cold until movement ceased and then the head was removed from the thorax. The ovaries were dissected into 1 × PBS (Phosphate buffered Saline) under a dissecting microscope. As the ovarioles were separated and the intima (external membrane on each ovariole) peeled off with fine forceps, they were placed in a microcentrifuge tube with 1 × PBS on ice. Once the ovarioles were separated and peeled, the samples were fixed in 1:1 heptane:4% formaldehyde in 1 × PBS for 15 minutes, and then rinsed in 1 × PTx (PBS + 0.1% Triton X-100) 3 times. Ovaries were either used immediately or stored in 100% methanol in the dark at −20°C.

### Identification of honeybee marker genes

Candidate genes were selected based on their developmental functions in mainly *Drosophila,* but also previous studies in *Apis* and other Hymenopterans. See Table 1 for further details. Honeybee orthologues were initially identified by reciprocal best Blastp (Altschul *et al*. 1990) hit and then further analyzed using Bayesian phylogeny techniques, using MrBayes 3.2.7a (Ronquist *et al*. 2012). Model of amino acid evolution used in these analyses was chosen after initial experiments using mixed models identified the most appropriate model. Castor, dpp, tapas, and hh were analyzed with a Whelan and Goldman model (Whelan and Goldman 2001), Traffic jam, Mad6, unzipped, and eyes absent with a Jones model (Jones *et al*. 1992), ova with a Dayhoff model (Dayhoff *et al*. 1978) and Bark beetle with a Blosum model (Henikoff and Henikoff 1992).

**Table 1.** Potential markers for somatic vs germline fate that were tested in this study.

Gene name	Predicted germline or somatic?	Expression in *Drosophila* ovary	Expression in other insect ovaries	References
*Vasa*	Germline	Germline	Germline (all holo- and hemimetabolous insects examined)	For examples see (Lasko and Ashburner 1988; Nakao 1999; Chang *et al*. 2002, 2004; Dearden 2006; Mito *et al*. 2008; Tanaka and Hartfelder 2009; Lynch *et al*. 2011; Cao *et al*. 2012; Ewen-Campen *et al*. 2012, 2013; Wang *et al*. 2020; Chung and Shigenobu 2022)
*Nanos*	Germline	Germline	Germline (all holo- and hemimetabolous insects examined)	For examples see (Wang *et al*. 1994; Chang *et al.* 2003, 2004; Adelman *et al*. 2007; Juhn *et al*. 2008; Zhao *et al*. 2008; Khila and Abouheif 2009, 2010; Ewen-Campen *et al*. 2012; Ogaugwu and Wimmer 2013; Hou *et al*. 2021; Chung and Shigenobu 2022; Huang *et al*. 2022)
*Bark-beetle*	Germline	Absent	Germline (*Nasonia*)	*Drosophila* (Li *et al*. 2022) *Nasonia* (Quan *et al*. 2019)
*Ovo*	Germline	Germline	Not clear in *Bombyx*	*Drosophila* (Mével-Ninio *et al*. 1991), *Bombyx* (Xue *et al*. 2014)
*Mad6*	Germline	Germline stem cells	No data	*Drosophila* (Song *et al*. 2004)
*Traffic-jam (tj)*	Somatic	Somatic cells in contact with the germline	No data	*Drosophila* (Li *et al*. 2003)
*Castor*	Somatic	Follicle cells	No data	*Drosophila* (Chang *et al*. 2013; Johnston *et al.* 2016)
*Eyes-absent (eya)*	Somatic	Follicle cells	Follicle cells (*Tribolium*)	*Drosophila* (Wang *et al*. 2007; Chang *et al*. 2013; Johnston *et al.* 2016) *Tribolium* (Bäumer *et al*. 2012)
*Decapentaplegic (dpp)*	Somatic	Follicle cells	Follicle cells (*Ceratitis capitata*)	*Drosophila* (Song *et al*. 2004; Vreede *et al*. 2013) *Ceratitis* (Vreede *et al*. 2013)
*Hedgehog (hh)*	Somatic	Terminal filament and cap cells	No data	*Drosophila* (Sahai-Hernandez and Nystul 2013)
*Unzipped*	Somatic	Described as somatic cells of the ovary	No data	*Drosophila* (Li *et al*. 2022)

### Hybridization chain reaction

Stored ovaries were rehydrated over a methanol series: 75%, 50%, 25% methanol/PTw (PBS + 0.1% Tween 20), and then rinsed 3 times in PTw for 5 minutes at each step. Rehydrated, or freshly fixed ovarioles were permeabilized in PTx for 2 hours at room temperature on a nutator.

Samples were prehybridized in 500 μL of 30% probe hybridization buffer (2.4 M Urea, 5 × sodium chloride sodium citrate (SSC), 9 mM citric acid (pH 6.0), 0.1% Tween 20, 50 μg/mL heparin, 1 × Denhardt's solution, 10% dextran sulfate), for 30 minutes at 37°C. Urea was used instead of formamide. Four hundred μL of the hybridization buffer was removed and the probes were added to the remaining 100 μL of hybridization buffer and samples at 37°C in one of the following concentrations: 2 μL of 1 μM probe (*nanos, castor*), 2 μL of 2 μM (odd and even) probe (*vasa*), 4 μL of 1 μM probe (*Tapas, bb, tj, ovo, eya*), 10 μL of 1 μM probe (*mad6, dpp, hh*), or 10 μL of 2 μM probe (*unzipped)* and the sample was incubated overnight (14–18 hours) to 5 days at 37°C. The probes were washed 4 × for 15 minutes each with 200 μL of probe wash buffer (2.4 M Urea, 5 × SSC, 9 mM citric acid (pH 6.0), 0.1% Tween, 50 μg/mL heparin) at 37°C and then washed 3 × for 5 minutes each with 500 μL of 5 × Standard Saline Citrate + Tween (SSCT) (5X SSC, 0.1% Tween 20) at room temperature.

Samples were incubated in 500 μL of amplification buffer (5X SSC, 0.1% Tween 20, 10% dextran sulfate) for 30 minutes at room temperature, while the hairpins were prepared by snap cooling 2 μL, of a 3 μM stock (6 pmol in 100 μL of buffer), per hairpin (keeping h1 and h2 separate)—heating to 90°C for 90 seconds, and cooling to room temperature in the dark for 30 minutes. Amplification buffer was removed and the snap-cooled hairpins (h1 and h2) were added in 100μL of amplification buffer at room temperature. The pre-amplification buffer was replaced with the hairpin mixture, and the samples were incubated overnight (14–18 hours) at room temperature in the dark.

The hairpins were removed by washing the samples with 500 μL of 5 × SSCT at room temperature for 5 minutes × 2, 30 minutes with 0.5 μL of DAPI [4′, 6-Diamidino-2-Phenylindole, Dihydochloride [D1306]] per 1 ml 5 × SSCT, 30 minutes in 5 × SSCT, and then a further 5 minutes in 5 × SSCT, all with gentle rocking. The sample was rinsed 3 × PTw and then stored in 70% ultrapure glycerol at 4°C in the dark.

### Immunohistochemistry

Ovarioles were used immediately after fixation for immunohistochemistry.

The fixed ovarioles were left in PTx for 2 hours on a rocker for permeabilization. Then they were put in 500 μL blocking solution, PBTB (Phosphate buffered block solution) (PBS +0.1% Triton X-100 + 5% normal goat serum + 0.2% Bovine Serum Albumin) for 30 minutes on the rocker at room temperature, or overnight in the fridge. The ovarioles were then put in primary antibody solution ((1:200 α-mouse-ph3 (Anti-Histone H3 (phospho S10) antibody [abcam 14955]): PBTB) overnight in the dark in the fridge.

The antibody solution was replaced with PTx and washed 4 × on the rocker for 15 minutes and the ovarioles were reblocked in blocking solution (PBTB) for 30 minutes. The blocking solution was replaced with a secondary antibody solution (1:1,000 goat-α-mouse 488[A11001]:PBTB) and left in the fridge overnight. The antibody solution was replaced with PTx and washed 4 × on the rocker in the dark for 15 minutes. The tissue was then counterstained with phalloidin (either Invitrogen Alexa Fluor488 phalloidin [A12379], or Invitrogen Alexa Fluor555 phalloidin [A34055]) (3 μL of a 66 μM solution per 100 μL PTx) for 15 minutes on a rocker. The samples were rinsed with PTx and then counterstained for DAPI (0.5μL per 1 mL of PTx) for 10 minutes. The tissue was finally rinsed 3 × with PTx and then stored in 70% glycerol in the fridge, in the dark until imaging.

### EdU staining

Queen bees were removed from the hive and carefully pushed, abdomen first, using a cotton wool pad, into a microcentrifuge tube modified with holes made in the bottom. A Hamilton 50 μL syringe fitted with a steel 22 s needle with a beveled tip was inserted shallowly between the tergites of the 4th and 5th segment dorsally and used to inject 8–10 μL of 10μM EdU (16–20 pmol), or until the abdomen visibly expanded. The queen was then put in a queen cage and returned to the hive to resume laying for either 24 hours or 48 hours. After the laying period, the queen was euthanized, and the ovaries were dissected, separated, and fixed as described above. Fixed ovarioles were used immediately, undergoing permeabilization in PTx for an hour on the rocker at room temperature. The tissue was soaked in PBTB for at least 30 minutes at room temperature to block nonspecific binding.

Tissue was treated and stained using the instructions of the Click-iT EdU cell proliferation kit (Thermofisher). The tissue was counterstained with DAPI (0.5 μL DAPI (10 mg/mL) per 1 mL PTx) for 15 minutes, before being rinsed 3 × with PTx. Finally, the samples were stored in 70% glycerol in the dark at 4°C.

### EdU staining longevity

We performed EdU injections across different periods of incubation (2 hours, 8 hours, and 4 days (∼96 hours)), on 2 different queens per time-period and stained as above for EdU incorporation in ovarioles. Ten ovarioles were isolated from each queen and completely imaged.

### Microscopy

Confocal microscopy was performed using an Olympus FV3000 confocal microscope. Germaria for hybridization chain reaction (HCR), phospho-Histone H3, and EdU imaging were imaged using 70–90 slices per stack depending on the thickness of the specimen. In all cases, the number of slices and depth was optimized using the FV3000 software to capture all information in the stack. Images were processed using either FIJI (Schindelin *et al*. 2012) or Icy (De Chaumont *et al*. 2012), with all projections shown being maximum intensity. All raw data images are available at Zenodo (10.5281/zenodo.8084467).

### Polyfusome counting and statistics

Ovaries were used fresh and stained with phalloidin (either Invitrogen Alexa Fluor555 phalloidin [A34055] or Invitrogen Alexa Fluor555 phalloidin [A34055]) and DAPI as described above. Images were taken on an Olympus FV3000 confocal microscope, of the early germarium encompassing each set of polyfusomes. Polyfusomes were counted blind by 3 staff experienced in insect ovary microscopy.

Means were compared between the older queen group and the younger queen group using an unpaired t.test in python using SciPy 1.7.0 stats.ttest_ind function, omitting NaN (Virtanen *et al*. 2020). Counts were made into boxplots based on each individual counter's (FNJ, FNP, and FNG) individual scores, and also in a boxplot summarizing the mean of all of the counts (FNM).

## Results

### High resolution structure of the queen honeybee ovariole

**Fig. 1.**
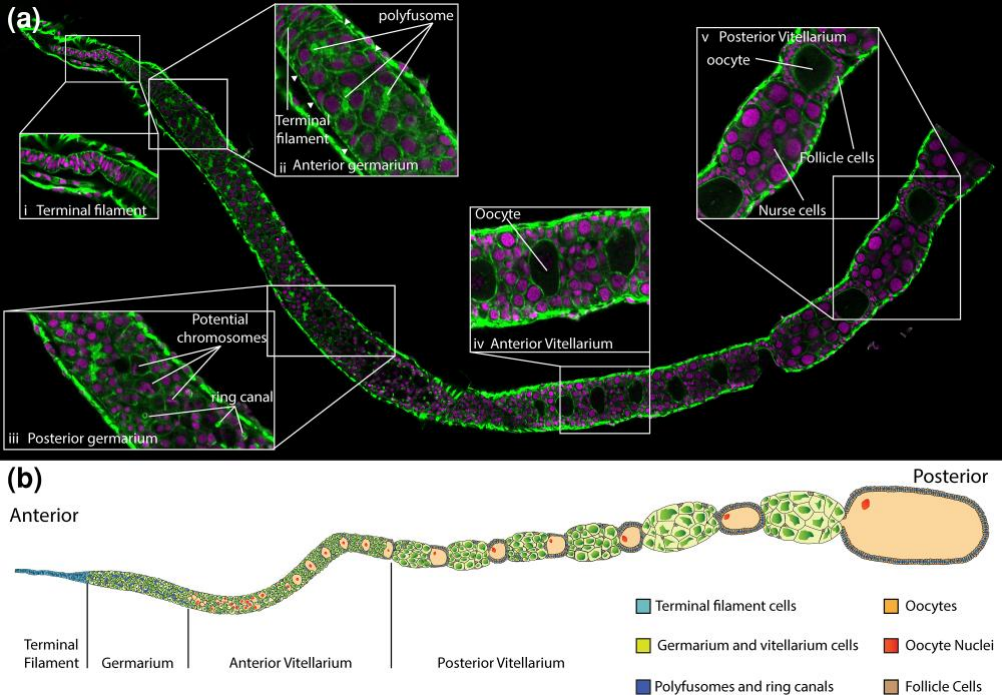
Ultrastructure of the honeybee queen ovariole. a) Composite confocal micrograph of a single honeybee queen ovariole stained with DAPI (purple) and Phalloidin (Green). Insets show key regions of the ovariole. i) Terminal filament showing flattened cells and nuclei ii) Anterior germarium, showing the transition from the flattened cells of the terminal filament to rounded cells and nuclei and polyfusomes staining with phalloidin. Arrowheads indicate single cells on the outside of the germarium, potentially follicle cells. iii) Posterior germarium, where polyfusomes disappear and are replaced by ring canals (circular phalloidin stained structures, in the same region we see DAPI stained chromosomes in a small patch of cells indicating cell division is occurring. iv) Anterior vitellarium. Here clear oocytes (marked by large size and very faint, or absent nuclear staining), line up in the ovariole. v) Posterior vitellarium, the oocyte and nurse cell group are separated, and follicle cells form a columnar epithelium around the oocyte. The oocyte expands. b) Cartoon of ovariole structure.

Confocal microscopy of DAPI and Phalloidin stained honeybee queen ovarioles (Fig. 1) is consistent with previous studies (Dearden 2006; Aamidor *et al*. 2022) and the morphology of other insect ovarioles. At the anterior end of the ovariole is the terminal filament, made up of initially a single, and then a double, stack of cells with flattened nuclei (Fig. 1). These cells appear to separate into a sheath of follicle cells around the germarium (arrowheads in inset Fig. 1). The germarium itself is populated by groups of 8 cells joined by a polyfusome, which stains strongly with phalloidin (Fig. 1). The posterior of the germarium is marked by the absence of polyfusomes and the appearance of ring canals, also strongly staining with phalloidin. At this point, in some cells, potentially condensed chromosomes can be seen (marked in inset Fig. 1), suggesting cell divisions of some sort are occurring in this region. Presumptive oocytes become larger than the surrounding cells and are surrounded by ring canals (Fig. 1). Once the ring canals cease, and the young oocytes are surrounded by follicle cells and move into separate chambers from their attached nurse cells, the cells are in the early vitellarium (Fig. 1).

Young oocytes become larger, and separate out into a single line (as in other hymenoptera (Eastin *et al*. 2020)) (Fig. 1). Finally the oocyte and nurse cells, still attached, are in separate chambers, the oocyte surrounded by follicle cells and filling with yolk granules. The oocyte continues to grow until the nurse cells collapse and dump their cytoplasm into the oocyte, releasing it for laying (Fig. 1). The entire ovariole is surrounded by a tough membrane, the intima.

**Fig. 2.**
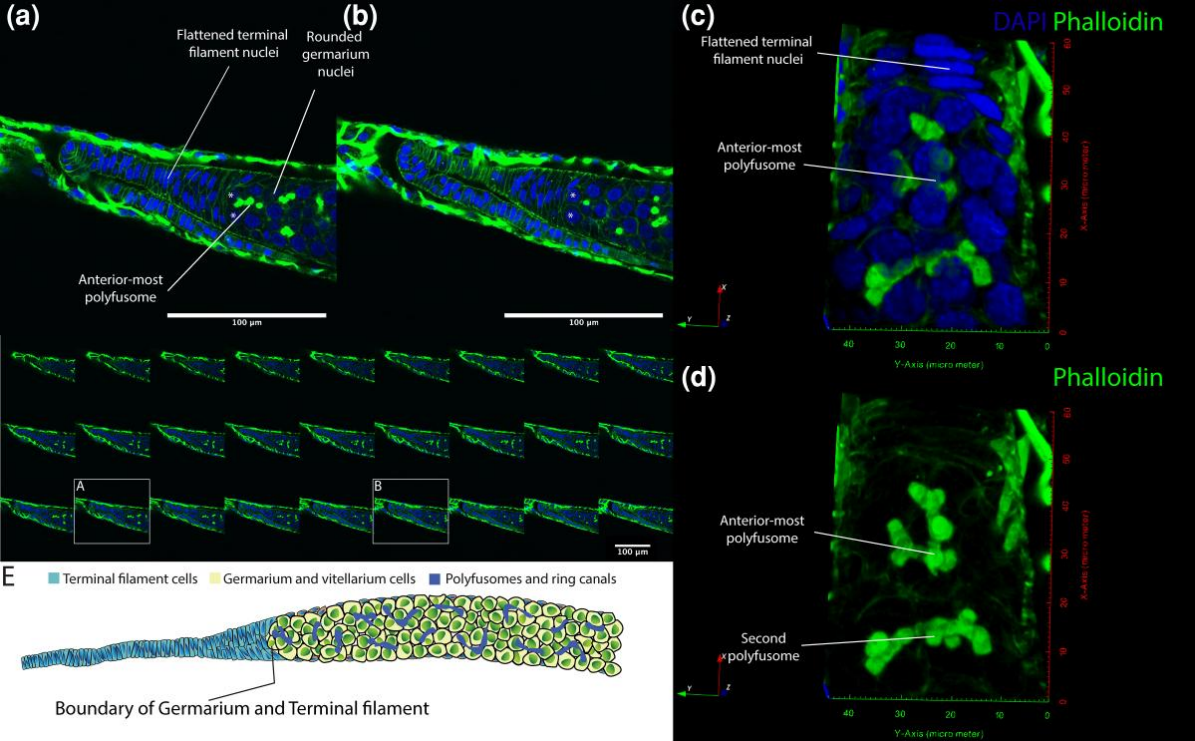
a) and b) single confocal sections across the boundary of the terminal filament and the germarium isolated from the montage of sections below. DAPI staining of nuclei (blue), and phalloidin staining (green) show the boundary between the flattened cells of the terminal filament and the rounded cells of the germarium. Germarium cells are joined by a polyfusome (staining with phalloidin). The 2 closest rounded cells to the terminal filament (marked with asterisks in a and b) are connected to surrounding cells by a polyfusome (most clearly shown in a). c) 3D projection through the boundary of the terminal filament and germarium, DAPI staining in blue, Phalloidin in green. The flattened nuclei of the terminal filament are marked and the most anterior polyfusome. d) The same projection as in c shows only phalloidin staining. Two branched polyfusomes are indicated. The images in c and d are shown in Movie form in Supplementary Movies 1 and 2 to better show the polyfusome structure. e) Cartoon of the boundary of the terminal filament and germarium are drawn to show no germarium cells that are not attached to a polyfusome, even at the most anterior end of the germarium.

One key question is the presence of single germline stem cells which has been predicted by several authors (Duncan *et al*. 2016; Hartfelder *et al*. 2018; Aamidor *et al*. 2022). Such germline stem cells, to follow the *Drosophila* model, would sit at the anterior end of the germarium, dividing as necessary. To search for the presence of these cells we focussed our scans on the anterior end of the germarium, attempting to determine if there is a population of single cells that may act as germline stem cells (Fig. 2). In none of these scans could we find single cells that did not have the morphology of terminal filament cells (Fig. 2c), or were not attached, via a polyfusome (Fig. 2d), to a rosette of 8 cells (See also Supplementary Movies 1 and 2).

### Somatic and germline cells in the honeybee ovary

To determine which cells in the honeybee ovariole are germline, and which are somatic, we used hybridization chain reaction *in-situ* hybridization (Choi *et al*. 2016, 2018) to examine the expression of putative germline and somatic marker genes gleaned from the insect literature. Table 1 shows the genes we tested as potential markers and the evidence for their expression in each cell type.

**Fig. 3.**
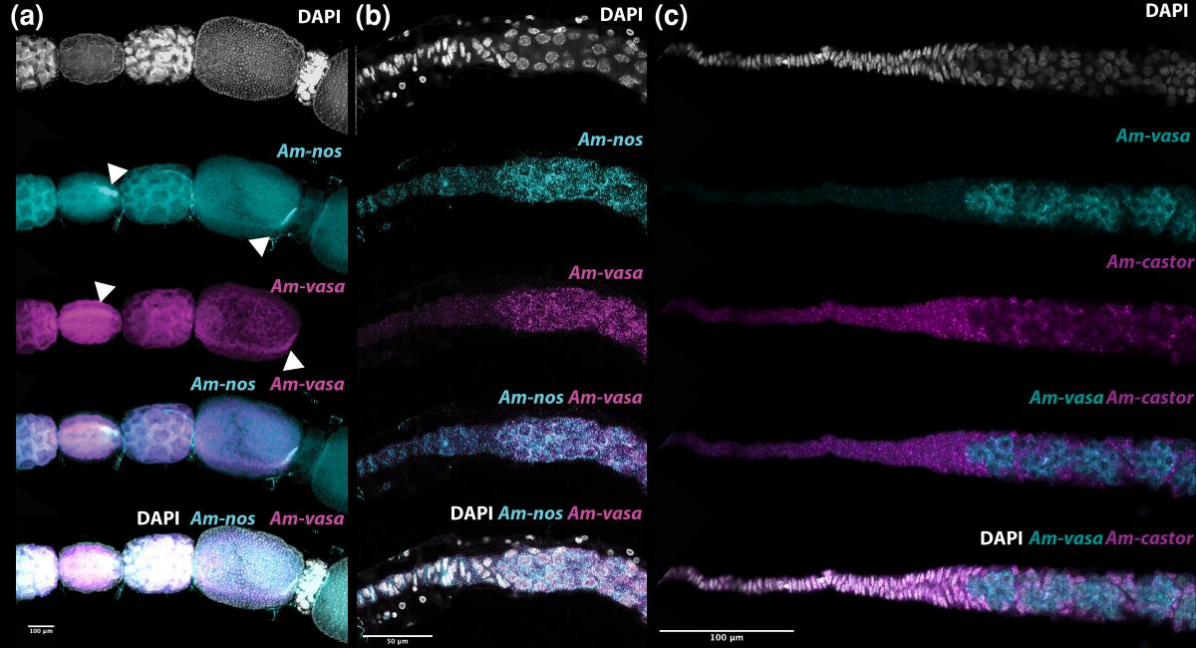
HCR in-situ hybridization imaging of germline and somatic markers in honeybee queen ovarioles. a) Vitellarium region of an ovariole stained for *Am-nanos* (cyan), *Am-vasa* (magenta), and DAPI (gray). Expression of these conserved germline markers is consistent with previous publications(Dearden 2006) with both germline markers expressed in nurse cells and the oocyte, with a concentration of RNA in a stripe down 1 side of the oocyte (arrowheads in a). There is no expression in follicle cells around the nurse-cell cluster or around the oocyte. b) Germarium and terminal filament region of the ovariole stained with *Am-nanos* (cyan), *Am-vasa* (magenta), and DAPI (gray). Both *Am-nos* and *Am-vasa* are expressed in the rounded germarium cells right up to the boundary with the terminal filament. No expression of either gene is present in the terminal filament. c) Germarium and terminal filament region of the ovariole stained with *Am-vasa* (cyan), *Am-castor* (magenta), and DAPI (gray). *Am-castor* expression is present in the terminal filament and follicle cells around the outside of the ovariole. Germline cells marked by *Am-vasa* are mutually exclusive to cells expressing *Am-castor*, implying *Am-castor* marks somatic cells in the ovary. As *Am-castor* is likely a somatic marker in the ovary (Chang *et al*. 2013; Johnston *et al.* 2016) this implies that no germline cells are present in the terminal filament (see also Supplementary Fig. 2 and Supplementary Movies 3–4).

RNA expression of *vasa* and *nanos* has long been established as germline markers in many animals (for examples see Nakao 1999; Castrillon *et al*. 2000; Tanaka *et al*. 2000; Tsunekawa *et al*. 2000; Chang *et al*. 2002, 2004; Dearden *et al*. 2003; Sagawa *et al*. 2005; Mito *et al*. 2008) including honeybees (Dearden 2006). The expression of both these genes has been examined in honeybee embryos showing that germline cells can be identified in presumptive ovary tissue in late honeybee embryos (Dearden 2006). RNA expression from these genes has also been examined in the ovary using histochemical methods, which did not produce a clear definition of the germline in the germarium (Dearden 2006). Using more sensitive HCR techniques (Choi *et al*. 2016, 2018) (Fig. 3) we found that both *vasa* and *nanos* expression are present in all the cells of the germarium-excepting the individual follicle cells on the outside of the germarium, and the cells of the terminal filament.

In the vitellarium, all cells but follicle cells stain strongly for *vasa* and *nanos* RNA (Fig. 3), and, as the oocyte matures, the RNA of both genes comes to be located in a streak down one surface of the oocyte from the oocyte nucleus in the anterior, to the posterior of the oocyte (Arrowheads in Fig. 3, as previously reported (Dearden 2006)).

**Fig. 4.**
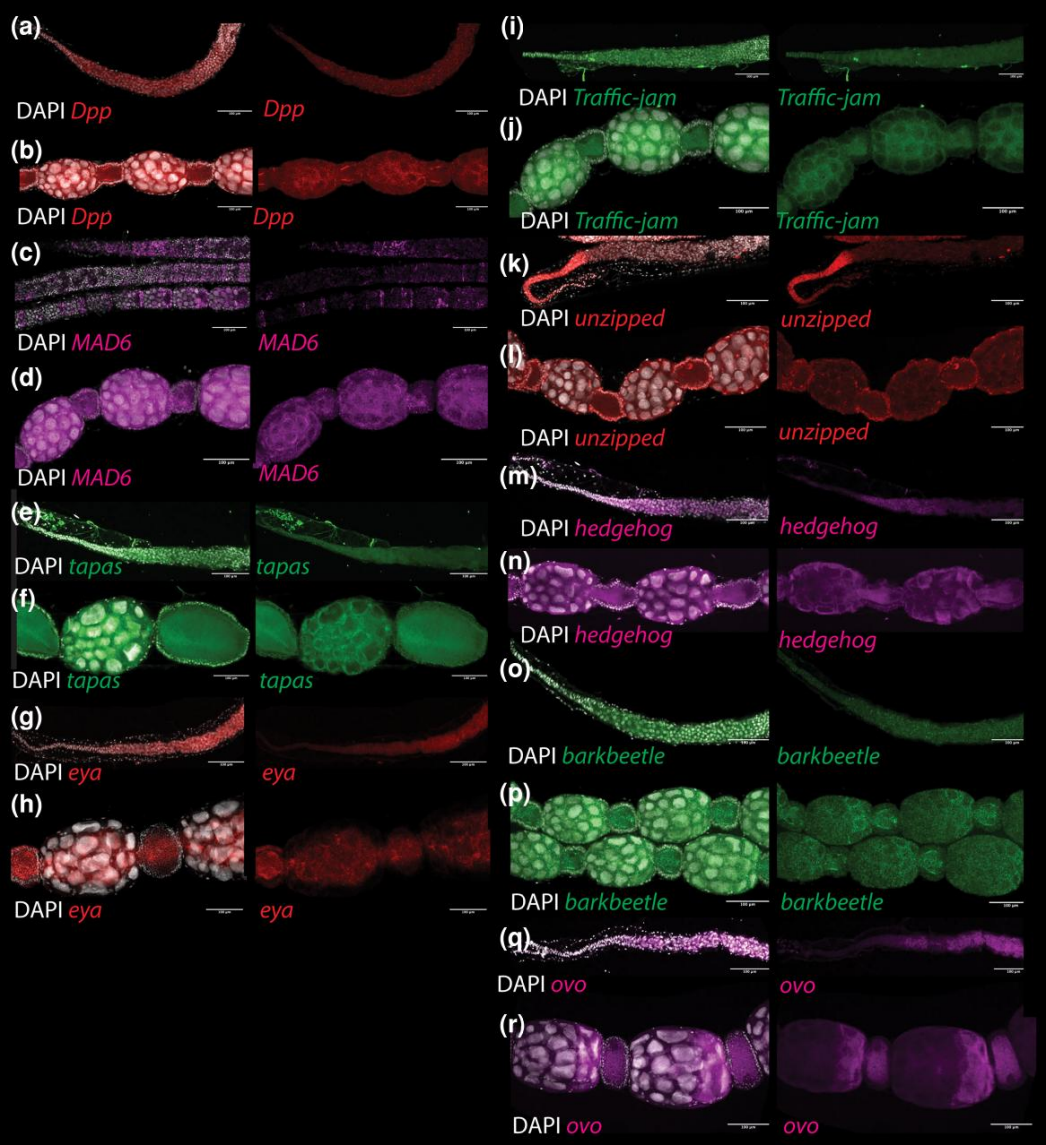
RNA in-situ patterns of ovary expressed genes in honeybee ovaries. Ovarioles are shown double-stained with DAPI and single-stained for the gene of interest. a) Expression of *dpp* in terminal filament and germarium, and b) vitellarium. Expression of *mad6* in c) terminal filament and germarium, and d) vitellarium. Expression of *tapas*in e) terminal filament and germarium, and f) vitellarium. Expression of *eya* in g) terminal filament and germarium, and h) vitellarium. Expression of *traffic-jam* in i) terminal filament and germarium, and j) vitellarium. Expression of *unzipped* in k) terminal filament and germarium, and l) vitellarium. Expression of *hedgehog* in m) terminal filament and germarium, and n) vitellarium. Expression of *barkbeetle* in o) terminal filament and germarium, and p) vitellarium. Expression of *ovo* in q) terminal filament and germarium, and r) vitellarium.

A few of our other potential markers showed similar gene expression in the germarium, including *dpp*, *ovo*, *eya*, and *hh* (Table 1 and Fig. 4). *Ovo*, in particular, is expressed in very similar regions to *vasa* and *nanos*, making it a useful germline marker. Other potential markers, such as *mad6*, also have an expression in somatic follicles cells in *Apis* (Fig. 4).

Somatic markers in the ovary have not been reported in insects outside of *Drosophila*, so we tested a panel of 6 markers, known to have somatic expression in *Drosophila*, to identify potential somatic cell markers. The honeybee ortholog of *castor*, a somatic marker in *Drosophila*, is strongly expressed in the terminal filament, and in follicle cells in the ovary; an exact inverse of the expression of *vasa* and *nanos* (Fig. 3 and Supplementary Fig. 1 and Supplementary Movies 3 and 4). In the germarium, *castor* is expressed in individual cells on the outside of the germarium. These cells, in the early germarium, appear to separate clusters of *vasa and nanos-*positive cells from each other (Supplementary Fig. 1 and Movies 3 and 4). We interpret these to be follicle cells that lie on the outside of the germarium. That they express a somatic marker and do not germline markers, implies these are not germline cells.

*Unzipped* RNA shows a similar pattern of expression to *castor* in honeybees (Table 1 and Fig. 4). In honeybees, *dpp*, *tj*, *hh*, and *eya* all appear to have expression in some cells that are germline as well as somatic cells in the ovary (Table 1 and Fig. 4), making them poor markers of the somatic cell population.

Identification of germline and somatic markers in the ovaries indicates that in honeybees, no germline cells are present in the terminal filament of the ovariole (Supplementary Fig. 2), as has been suggested by previous authors (Gutzeit *et al*. 1993). We have been unable to image polyfusomes alongside *in-situ* staining, as these 2 staining techniques seem incompatible. Taking our staining evidence together, however, the first germline cells of the ovary, as shown by the presence of *vasa* and *nanos* expression, and lack of *castor* expression, are 8 cell clusters (Fig. 3, Fig. 2), and these are in the same location and have that same appearance as those joined by a polyfusome stained with phalloidin (Fig. 2), in the germarium. This implies these are the stock of germline cells in the ovariole. We can identify no single *vasa*/nanos positive cells in the germarium, and in this region, we find 8-cell clusters joined by polyfusomes (Fig. 2). In honeybee queen ovarioles, it appears that the germline begins as clusters of 8 cells, physically joined by the polyfusome (Fig. 2 and 3), located in the germarium.

Our data imply that the germline cells in the ovary, detected by germline markers (Fig. 3) arranged as 8 cell clusters (rosettes in previous literature (Büning 1994)) joined by polyfusomes (Fig. 2). In these experiments we can detect no single, germ stem-cells in the ovary, leads us to question how the high fecundity of queen bees is achieved. Are the ovaries of queen bees already populated with all the germline precursors, as 8-cell clusters, that are needed after eclosion? Or are these 8 cell clusters capable of dividing? By examining markers of cell division, we aimed to rule out the presence of single-dividing germline stem cells, and determine if the 8 cell clusters of germline cells can divide, indicating the possibility that they may be germline precursors.

### Cell division in the honeybee ovariole

**Fig. 5.**
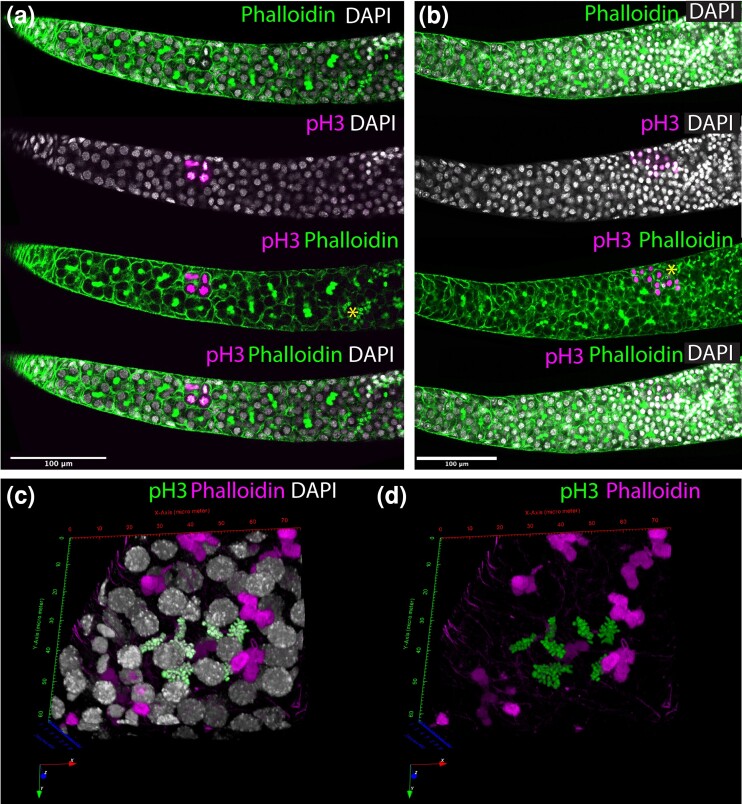
Immunohistochemistry for phospho-histone H3 in the germarium of honeybee ovarioles. a) Single confocal section of the anterior end of a germarium stained for phalloidin (green) and phospho-histone H3 (marking dividing cells (magenta)). In the anterior germarium 1 set of 8 cells (4 visible in this section) stains strongly with phospho-histone H3 (and can be seen to have metaphase chromosomes in DAPI). An asterisk marks the boundary between polyfusomes and ring canals. b) Single confocal section of the posterior end of a germarium stained for phalloidin (green) and phospho-histone H3 (magenta). Phospho-histone H3 positive cells can be seen at the boundary of the germarium, where polyfusomes give way to ring canals (boundary marked with an asterisk). c) 3D projection of a confocal series through a Phospho-histone H3 labeled cluster of cells, stained for DNA, using DAPI (gray), phalloidin (magenta), and Phospho-histone H3 (green). d) The same projection as in C, but showing only phalloidin (magenta) and Phospho-histone H3 (green). Movies of 3D rotation of these sections are shown in Supplementary Movies 5–8.

To begin to determine where cell division may be occurring in the germline of actively laying queen honeybee ovarioles we used immunohistochemistry against the mitosis marker phospho-Histone H3 (Harsh *et al*. 2020) to identify cells undergoing division. Cells undergoing division in the germarium of ovarioles appear in 2 places in each ovariole (Fig. 5). Under these conditions, we do not see cell divisions in 8-cell clusters at the boundary of the terminal filament and the germarium.

The first germarium region where mitosis is occurring is approximately halfway between the terminal filament and the posterior end of the germarium. Cells are often in mitosis in this area but always in clusters. These clusters, made up of 8 cells, undergo mitosis in a synchronized way, producing 2 daughter 8-cell clusters from the original one. This data indicates that, despite the germline cells being linked by polyfusomes into 8-cell clusters, they are still able to divide, increasing the number of germline cell clusters in each ovariole (Fig. 5, Supplementary Movies 5–8). That we catch considerable numbers of clusters in M-phase (19/32, Supplementary Table 1) using phospho-Histone H3 implies that this phase is quite long.

The second region is in the posterior germarium where polyfusomes are replaced by ring canals (Fig. 5). This is the last synchronous mitotic division of the full cluster, after which the oocyte becomes specialized and will undergo meiosis. We are yet to find a suitable meiosis marker in honeybees to confirm this.

We did not find dividing single cells at the base of the terminal filament, as would be expected for a set of germline stem cells as in *Drosophila.* We also do not find 8-cell clusters dividing in this region, though it is possible that clusters located here divide in environmental conditions not produced in our experiments. We can find no single non-terminal filament cells that stain with *vasa* or *nanos* and divide frequently, implying the arrangement of single germline stem cells seen in *Drosophila* are absent from the honeybee ovariole.

As our phospho-Histone H3 staining indicates that the 8-cell clusters in the germarium can divide, these have the characteristics, expressing germline markers and dividing frequently, of germline precursors of the ovary, and are thus key to the long-term fecundity of honeybees. In our images, polyfusomes of dividing clusters are fainter when stained with phalloidin than those not dividing (Fig. 5d) implying synchronous division may be associated with some dissolution of the polyfusome.

### Imaging germline division in honeybee germaria

To assess if there are other potential germline cells in the germarium that are more rapidly dividing, such that we can’t detect them with phospho-Histone H3, we used EdU staining. EdU, a nucleotide analog similar to BrdU, is incorporated into newly synthesized DNA in the S phase of cell division and remains detectable after cell division has occurred. While it would be convenient to observe these divisions in tissue culture, we were concerned that ovarioles would react differently when removed from queen bees. To remove this concern we injected EdU into actively laying queens during spring and summer, allowed them to continue laying, and then euthanized queens and examined their ovaries.

**Fig. 6.**
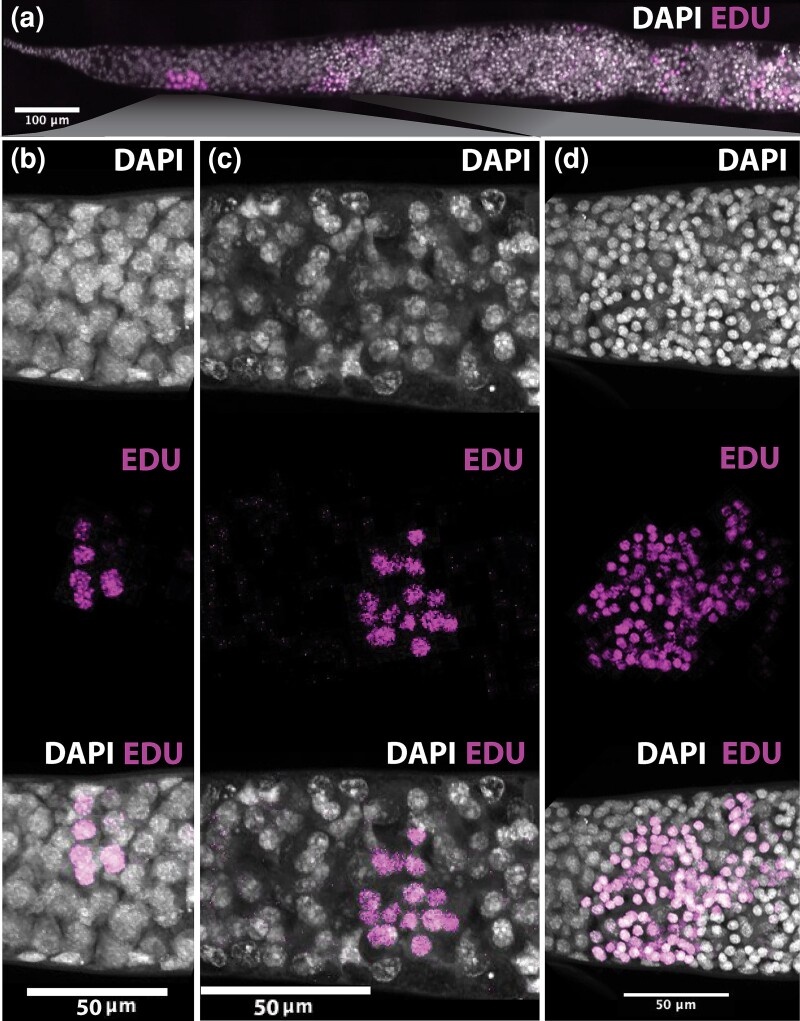
Detection of dividing cells using EdU after 24 hours of exposure. a) Terminal filament, germarium, and anterior vitellarium of an ovariole from a queen bee injected with EdU, left to lay in a hive and fixed and stained after 24 hours. DAPI stained nuclei in gray and EdU, indicating DNA replication in the 24 hours (and thus cell division), in magenta. B) EdU staining in a cluster of 6–8 cells in the anterior of the germarium. c) A cluster of EdU marked 16 cells in the anterior of the germarium. d) A large cluster of EdU-marked cells at the boundary of the germarium and anterior vitellarium. e) Detection of dividing cells using EdU after 48 hours of exposure. EdU staining in 2 clusters of cells in the anterior of the germarium and a large cluster of EdU marked cells at the boundary of the germarium and anterior vitellarium.

To determine when the appropriate time to assess cell divisions might be, we performed a pilot experiment to determine how quickly EdU is taken up and how long it remains in the ovary. We found that cells that have taken up EdU into the nucleus are detectable 2 hours after injection and that cells still take up EdU after 4 days of incubation (Supplementary Fig. 3). Given this range of detectable activity, we examined EdU-stained ovarioles at 24 and 48 hours after injection (Fig. 6).

Examining ovarioles labeled and dissected after 24 hours indicates that, under these experimental conditions, clusters of dividing cells in the germarium appear in only 2 places, as with phospho-Histone H3 staining. Clusters of cells are present midway along the germarium, and at the boundary of the germarium and anterior vitellarium (Fig. 6). Dissecting after 48 hours (Fig. 6) identifies no other clusters of dividing cells. In each case, cells labeled with EdU are in clusters of 8 (rarely), 16, 24, or 36 (see Supplementary Table 2). This implies that once a cluster divides, it can divide again.

In our experiments, we see no evidence of single-dividing cells in the germarium adjacent to terminal filament cells. It is possible that such cells could be present, but be dividing very slowly. Given we cannot identify any germline cells in this region that are not next to a fusome (Fig. 2), it seems unlikely. The nongermline cells on the outside of the germarium do not appear to divide within the timeframe of this experiment, but do later in the vitellarium (Supplementary Fig. 3).

We cannot estimate how long it takes for the EdU injection to get to the ovary and how long the EdU remains in the reproductive system before it is completely used, how damaging the injection and handling is to queen reproduction (though all queens laid after injection), nor how much variation is added by local temperatures/weather conditions/hive placement, etc. Endoreplication in the nuclei of nurse cells is also common in insects and occurs in other hymenoptera (Eastin *et al*. 2020). EdU staining consistent with endoreplication was seen in these experiments, with nurse cell nuclei in the vitellarium occasionally staining strongly with EdU (data not shown). We cannot rule out endoreplication occurring earlier, but divisions in the germarium appear to produce 2 daughter 8-cell clusters, rather than some of those cells staining for EdU and not dividing. For these reasons, it is very difficult to use this data to determine the rate of cluster division.

Environmental influence on cluster division is very likely. This data was collected under normal summer laying conditions. Environmental conditions affect queen egg-laying such that queens do not always lay the maximum number of eggs possible every 24 hours, and indeed are reproductively inactive in cold weather. Our estimates are thus confounded by the significant environmental input into queen egg laying. While it is possible that the presence of 8-cell clusters, rather than single germline cells, in the adult ovary is related to environmental conditions, these clusters have been identified in larval ovaries (Hartfelder and Steinbrück 1997) implying they form before regulation of egg-laying occurs. To attempt to understand the long-term influence of 8-cell cluster division in queen ovaries, we compared the stock of germline cells in young and older queens.

### Older queens do not have fewer germ-cell clusters than first season queens

While our data indicate that 8-cell clusters can divide, it is not clear if the rate of that division is enough to maintain reproductive potential over the life time of queens. To determine if queen honeybees’ reproductive capacity is limited by the number of 8-cell clusters that form in their ovaries during larval development (Hartfelder and Steinbrück 1997), we counted the number of polyfusomes in the germaria of first season (labeled young) (3–10-month-old) vs second + season labeled old (1–2-year-old) laying queens. If 8-cell clusters are the germ-cell units in honeybees, polyfusome number is an estimate of reproductive capacity in an ovariole. Polyfusomes are easily detected by phalloidin staining, so ovarioles from queens were stained with phalloidin, and confocal stacks were made. Polyfusomes were scored blind and independently by 3 experienced researchers. All the old queens in this experiment were used in normal commercial beekeeping operations and maintained outdoors near Dunedin, New Zealand. Old queens had slightly higher, but not statistically significant (*P*-value 0.159), number of polyfusomes (μ = 23.63, σ^2^ = 3.81) compared to young queens (μ = 22.49, σ^2^ = 4.46) (Supplementary Fig. 4).

Our analysis shows no statistically significant difference in polyfusomes between young and old queens. This implies that the division of 8-cell clusters in the germarium occurs at a rate capable of replacing the stock of germline cells in queens over the long term. It seems likely that honeybee queens, at least over 1–2 years, do not run out of germline cells in their ovary, and queen reproductive failure is more likely due to other effects (such as sperm depletion or damage).

## Discussion

Honeybee queens, like queens in many eusocial species, have remarkable reproductive capacity. This capacity is required to populate bee colonies over long periods and must be exquisitely responsive to the environment. This reproductive capacity has led to changes in the structure of the ovaries of honeybees, the most obvious being the large number of ovarioles in each of the 2 ovaries. The reproductive capacity of honeybees underpins natural and managed pollination around the world and the hive-produces industry.

Our data indicate that, unlike the model system *Drosophila*, there is no single germline stem cells, as suggested by previous authors (Duncan *et al*. 2016; Aamidor *et al*. 2022), responsible for the production of oocytes located in the adult honeybee germarium. We cannot detect these cells in the structure of the ovary, nor do any such cells stain with germline markers, nor marked by phospho-histone H3, indicating active division, nor EdU, indicating division in at least 48 hours. Electron-microscopy (Gutzeit *et al*. 1993) and *in-situ* studies (Dearden 2006) have suggested that germline stem cells reside in the terminal filament of the honeybee ovary, but there are no cells in the terminal filament that stain for RNA from the germline markers *vasa* or *nanos*, and all these cells stain with our somatic marker *castor*. This arrangement is consistent with data from the polytrophic meroistic ovaries of other Hymenoptera (Büning 1994).

The germ-cell stock of the honeybee ovary appears then to be the collection of 8-cell clusters, linked by polyfusomes, which populate the germarium. These stain with the germline markers *vasa* or *nanos*, and are not somatic. This explains why bees have so many polyfusomes in each ovariole (average of 23) compared to *Drosophila*. In *Drosophila*, cystocytes with fusomes are a transient stage between germline stem cells and meiosis (McLaughlin and Bratu 2015). In honeybees, the 8-cell clusters, effectively the homologs of cystocyte clusters, are the germline cell population. These 8-cell clusters do not appear to form in the adult ovary but must be produced during larval (Hartfelder and Steinbrück 1997) and pupal development as the ovary forms, presumably from primordial germline stem cells which form late in embryogenesis (Dearden 2006). Given the germline cells in the honeybee ovary are arranged in clusters, joined by polyfusomes, it would be unremarkable to suggest that these cannot divide and that the complement of the clusters in honeybee ovaries represents ALL the reproductive capacity of a queen bee. Our data shows this is not the case. The germline cell clusters can, and do, divide. Examination of the germaria of old and young queens indicates that this rate of division is enough to maintain the reproductive potential of queens over their long lives. We find no sign of reproductive potential decline after 1 year of activity in queen bees, implying that, with good nutrition and a conducive environment, queen bees will remain highly reproductively active between seasons at least in their younger years.

How the conjoined clusters of germline cells achieve division with a polyfusome joining them is unknown, and will require more detailed observation of the division process with better markers for the polyfusome or live cell imaging. Why division appears to be limited to a site about halfway along the germarium is also unknown, suggesting perhaps a permissive environment that exists at that site. It is possible that these clusters next to the terminal filament cells actively divide only when the ovary is producing the large numbers of oocytes honeybee queens lay in ideal conditions (Allen 1955; Harbo 1986; Avni *et al*. 2014). It is also possible that these clusters break down into individual cells in poor conditions, as cystocytes can do in Drosophila ovaries (Kai and Spradling 2004), though the presence of such clusters in honeybee larval ovaries (Hartfelder and Steinbrück 1997) implies cluster formation occurs during larval development. Single germline cells may also reside at the terminal filament/germarium boundary in repressed or active worker honeybee ovaries (Duncan *et al*. 2016).

Our identification of the germline cell niche in honeybees allows us to better understand reproduction in this insect and begin to unpick the relationships between fecundity and environmental challenges. Our finding that these clusters of cells have the potential to divide may provide the background knowledge to develop new reproductive technologies for these economically important insects.

## Data Availability

The authors affirm that all data necessary for confirming the conclusions of the article are present within the article, figures, and tables. Supplementary Movies 1-8 are available at figshare: https://doi.org/10.25386/genetics.22697986. Contents of Supplementary Movies are described in the Supplementary Legends file. Raw data files are available at Zenodo (10.5281/zenodo.8084467).

## References

[iyad138-B1] Aamidor SE , Cardoso-Júnior CAM, Harianto J, Nowell CJ, Cole L, Oldroyd BP, Ronai I. Reproductive plasticity and oogenesis in the queen honey bee (Apis mellifera). J Insect Physiol. 2022;136(Jan):104347. doi:10.1016/j.jinsphys.2021.104347. 34902433

[iyad138-B2] Adelman ZN , Jasinskiene N, Onal S, Juhn J, Ashikyan A, Salampessy M, MacCauley T, James AA. Nanos gene control DNA mediates developmentally regulated transposition in the yellow fever mosquito Aedes aegypti. Proc Natl Acad Sci U S A. 2007;104(24):9970–9975. doi:10.1073/pnas.0701515104. 17548819 PMC1891237

[iyad138-B3] Allen MD . Observations on honeybees attending their queen. Br J Anim Behav. 1955;3(2):66–69. doi:10.1016/S0950-5601(55)80015-9.

[iyad138-B4] Altschul SF , Gish W, Miller W, Myers EW, Lipman DJ. Basic local alignment search tool. J Mol Biol. 1990;215(3):403–410. doi:10.1016/S0022-2836(05)80360-2. 2231712

[iyad138-B5] Avni D , Hendriksma HP, Dag A, Uni Z, Shafir S. Nutritional aspects of honey bee-collected pollen and constraints on colony development in the eastern Mediterranean. J Insect Physiol. 2014;69:65–73. doi:10.1016/j.jinsphys.2014.07.001. 25038311

[iyad138-B6] Bäumer D , Ströhlein NM, Schoppmeier M. Opposing effects of Notch-signaling in maintaining the proliferative state of follicle cells in the telotrophic ovary of the beetle Tribolium. Front Zool. 2012;9(1):15. doi:10.1186/1742-9994-9-15. 22866820 PMC3502128

[iyad138-B7] Büning J . The Insect Ovary. Dordrecht: Springer Netherlands; 1994.

[iyad138-B8] Cao G , Zhang Y, Xue R, Zhu Y, Wei Y, Zheng X, Gong C. Alternative splicing, expression patterns and promoter characters of vasa-like gene from the silkworm, Bombyx mori. Mol Biol Rep. 2012;39(5):5967–5976. doi:10.1007/s11033-011-1409-7. 22207176

[iyad138-B9] Castrillon DH , Quade BJ, Wang TY, Quigley C, Crum CP. The human VASA gene is specifically expressed in the germ cell lineage. Proc Natl Acad Sci U S A. 2000;97(17):9585–9590. doi:10.1073/pnas.160274797. 10920202 PMC16908

[iyad138-B10] Chang C , Dearden P, Akam M. Germ line development in the grasshopper Schistocerca gregaria: vasa as a marker. Dev Biol. 2002;252(1):100–118. doi:10.1006/dbio.2002.0840. 12453463

[iyad138-B11] Chang CC , Huang TY, Cook CE, Lin GW, Shih CL, Chen RP. Developmental expression of Apnanos during oogenesis and embryogenesis in the parthenogenetic pea aphid Acyrthosiphon pisum. Int J Dev Biol. 2003;53(1):169–176. doi:10.1387/ijdb.082570cc. 19123140

[iyad138-B12] Chang Y-C , Jang AC-C, Lin C-H, Montell DJ. Castor is required for Hedgehog-dependent cell-fate specification and follicle stem cell maintenance in Drosophila oogenesis. Proc Natl Acad Sci U S A. 2013;110(19):E1734–E1742. doi:10.1073/pnas.1300725110. 23610413 PMC3651482

[iyad138-B13] Chang C-C , Lee W-C, Cook CE, Lin G-W, Chang T. Germ-plasm specification and germline development in the parthenogenetic pea aphid Acyrthosiphon pisum: vasa and nanos as markers. Int J Dev Biol. 2004;50(4):413–421. doi:10.1387/ijdb.052100cc. 16525937

[iyad138-B14] Choi HM , Calvert CR, Husain N, Huss D, Barsi JC, Deverman BE, Hunter RC, Kato M, Lee SM, Abelin AC, et al Mapping a multiplexed zoo of mRNA expression. Development. 2016;143(19):3632–3637. doi:10.1242/dev.140137. 27702788 PMC5087610

[iyad138-B15] Choi HMT , Schwarzkopf M, Fornace ME, Acharya A, Artavanis G, Stegmaier J, Cunha A, Pierce NA. Third-generation in situ hybridization chain reaction: multiplexed, quantitative, sensitive, versatile, robust. Development. 2018;145(12):dev165753. doi:10.1242/dev.165753. PMC6031405 29945988

[iyad138-B16] Chung C , Shigenobu S. Reproductive constraint in the social aphid Ceratovacuna japonica: sterility regulation in the soldier caste of a viviparous insect. Insect Biochem Mol Biol. 2022;145(Jun):103756. doi:10.1016/j.ibmb.2022.103756. 35367587

[iyad138-B17] Church SH , de Medeiros BAS, Donoughe S, Márquez Reyes NL, Extavour CG. Repeated loss of variation in insect ovary morphology highlights the role of development in life-history evolution. Proc R Soc B Biol Sci. 2021;288(1950):20210150. doi:10.1098/rspb.2021.0150. PMC8097220 33947234

[iyad138-B18] Dayhoff M O, Schwartz RM, Orcutt BC. Atlas of Protein Sequence and Structure. Dayhoff MO, editor. National Biomedical Research Foundation; 1978. p. 345–352.

[iyad138-B19] Dearden P . Germ cell development in the honeybee (Apis mellifera); vasa and nanos expression. BMC Dev Biol. 2006;6(1):6. doi:10.1186/1471-213X-6-6. 16503992 PMC1388196

[iyad138-B20] Dearden P , Grbic M, Donly C. Vasa expression and germ-cell specification in the spider mite Tetranychus urticae. Dev Genes Evol. 2003;212(12):599–603. doi:10.1007/s00427-002-0280-x. 12536324

[iyad138-B21] de Chaumont F , Dallongeville S, Chenouard N, Hervé N, Pop S, Provoost T, Meas-Yedid V, Pankajakshan P, Lecomte T, Le Montagner Y, et al Icy: an open bioimage informatics platform for extended reproducible research. Nat Methods. 2012;9(7):690–696. doi:10.1038/nmeth.2075. 22743774

[iyad138-B22] Duncan EJ , Hyink O, Dearden PK. Notch signalling mediates reproductive constraint in the adult worker honeybee. Nat Commun. 2016;7(1):12427. doi:10.1038/ncomms12427. 27485026 PMC4976197

[iyad138-B23] Eastin KJ , Huang AP, Ferree PM. A novel pattern of germ cell divisions in the production of hymenopteran insect eggs. Biol Lett. 2020;16(5):20200137. doi:10.1098/rsbl.2020.0137. PMC7280034 32396789

[iyad138-B24] Ewen-Campen B , Jones TEM, Extavour CG. Evidence against a germ plasm in the milkweed bug *Oncopeltus fasciatus*, a hemimetabolous insect. Biol Open. 2013;2(6):556–568. doi:10.1242/bio.20134390. 23789106 PMC3683158

[iyad138-B25] Ewen-Campen B , Srouji JR, Schwager EE, Extavour CG. Oskar predates the evolution of germ plasm in insects. Curr Biol. 2012;22(23):2278–2283. doi:10.1016/j.cub.2012.10.019. 23122849

[iyad138-B26] Gutzeit HO , Zissler D, Fleig R. Oogenesis in the honeybee Apis mellifera: cytological observations on the formation and differentiation of previtellogenic ovarian follicles. Roux’s Arch Dev Biol. 1993;202(3):181–191. doi:10.1007/BF00365309. 28305996

[iyad138-B27] Harbo JR . Oviposition rates of instrumentally inseminated and naturally mated queen honey bees (hymenoptera: apidae). Ann Entomol Soc Am. 1986;79(1):112–115. doi:10.1093/aesa/79.1.112.

[iyad138-B28] Harsh S , Fu Y, Kenney E, Han Z, Eleftherianos I. Zika Virus non-structural protein NS4A restricts eye growth in Drosophila through regulation of JAK/STAT signaling. Dis Model Mech. 2020;13(4):dmm040816. doi:10.1242/dmm.040816. PMC7197722 32152180

[iyad138-B29] Hartfelder K , Steinbrück G. Germ cell cluster formation and cell death are alternatives in caste-specific differentiation of the larval honey bee ovary. Invertebrate Reprod Dev. 1997;31(1–3):237–250. doi:10.1080/07924259.1997.9672582.

[iyad138-B30] Hartfelder K , Tiberio GJ, Lago DC, Dallacqua RP, Bitondi MMG. The ovary and its genes—developmental processes underlying the establishment and function of a highly divergent reproductive system in the female castes of the honey bee, Apis mellifera. Apidologie (Celle). 2018;49(1):49–70. doi:10.1007/s13592-017-0548-9.

[iyad138-B31] Hegner RW . Studies on germ cells. IV. Protoplasmic differentiation in the oocytes of certain Hymenoptera. J Morphology. 1915;26(3):495–561.

[iyad138-B32] Henikoff S , Henikoff JG. Amino acid substitution matrices from protein blocks. Proc Natl Acad Sci U S A. 1992;89(22):10915–10919. doi:10.1073/pnas.89.22.10915. 1438297 PMC50453

[iyad138-B33] Hou Q-L , Chen E-H, Xie Y-F, Dou W, Wang J-J. Ovary-specific transcriptome and essential role of nanos in ovary development in the oriental fruit fly (Diptera: tephritidae). J Econ Entomol. 2021;114(2):947–958. doi:10.1093/jee/toab004. 33537732

[iyad138-B34] Huang Z , Tian Z, Zhao Y, Zhu F, Liu W, Wang X. MAPK signaling pathway is essential for female reproductive regulation in the cabbage beetle, Colaphellus bowringi. Cells. 2022;11(10):1602. doi:10.3390/cells11101602. 35626638 PMC9140119

[iyad138-B35] Jackson JT , Tarpy DR, Fahrbach SE. Histological estimates of ovariole number in honey bee queens, *Apis mellifera*. Reveal lack of correlation with other queen quality measures. J Insect Sci. 2011;11(82):1–11. doi:10.1673/031.011.8201. 21870968 PMC3398436

[iyad138-B36] Johnston MJ , Bar-Cohen S, Paroush Z, Nystul TG. Phosphorylated Groucho delays differentiation in the follicle stem cell lineage by providing a molecular memory of EGFR signaling in the niche. Development. 2016;143(24):4631–4642. doi:10.1242/dev.143263. 27836963 PMC5201033

[iyad138-B37] Jones DT , Taylor WR, Thornton JM. The rapid generation of mutation data matrices from protein sequences. Comput Appl Biosci. 1992;8(3):275–282. doi:10.1093/bioinformatics/8.3.275. 1633570

[iyad138-B38] Jordan A , Patch HM, Grozinger CM, Khanna V. Economic dependence and vulnerability of United States agricultural sector on insect-mediated pollination service. Environ Sci Technol. 2021;55(4):2243–2253. doi:10.1021/acs.est.0c04786. 33496588

[iyad138-B39] Juhn J , Marinotti O, Calvo E, James A. Gene structure and expression of nanos (nos) and oskar (osk) orthologues of the vector mosquito, Culex quinquefasciatus. Insect Mol Biol. 2008;17(5):545–552. doi:10.1111/j.1365-2583.2008.00823.x. 18828840 PMC3721150

[iyad138-B40] Kai T , Spradling A. Differentiating germ cells can revert into functional stem cells in Drosophila melanogaster ovaries. Nature. 2004;428(6982):564–569. doi:10.1038/nature02436. 15024390

[iyad138-B41] Khila A , Abouheif E. In situ hybridization on ant ovaries and embryos. Cold Spring Harb Protoc. 2009;2009(7):pdb.prot5250. doi:10.1101/pdb.prot5250. 20147215

[iyad138-B42] Khila A , Abouheif E. Evaluating the role of reproductive constraints in ant social evolution. Philos Trans R Soc B Biol Sci. 2010;365(1540):617–630. doi:10.1098/rstb.2009.0257. PMC2817144 20083637

[iyad138-B43] Kirilly D , Xie T. The Drosophila ovary: an active stem cell community. Cell Res. 2007;17(1):15–25. doi:10.1038/sj.cr.7310123. 17199109

[iyad138-B44] Klepsatel P , Gáliková M, De Maio N, Ricci S, Schlötterer C, Flatt T. Reproductive and post-reproductive life history of wild-caught *Drosophila melanogaster* under laboratory conditions. J Evol Biol. 2013;26(7):1508–1520. doi:10.1111/jeb.12155. 23675912

[iyad138-B45] Lasko PF , Ashburner M. The product of the Drosophila gene vasa is very similar to eukaryotic initiation factor-4A. Nature. 1988;335(6191):611–617. doi:10.1038/335611a0. 3140040

[iyad138-B46] Li MA , Alls JD, Avancini RM, Koo K, Godt D. The large Maf factor traffic jam controls gonad morphogenesis in Drosophila. Nat Cell Biol. 2003;5(11):994–1000. doi:10.1038/ncb1058. 14578908

[iyad138-B47] Li H , Janssens J, De Waegeneer M, Kolluru SS, Davie K, Gardeux V, Saelens W, David FPA, Brbić M, Spanier K, et al Fly cell atlas: a single-nucleus transcriptomic atlas of the adult fruit fly. Science. 2022;375(6584):eabk2432. doi:10.1126/science.abk2432. PMC8944923 35239393

[iyad138-B48] Lynch JA , Ozüak O, Khila A, Abouheif E, Desplan C, Roth S. The phylogenetic origin of oskar coincided with the origin of maternally provisioned germ plasm and pole cells at the base of the holometabola. PLoS Genet. 2011;7(4):e1002029. doi:10.1371/journal.pgen.1002029. PMC3084197 21552321

[iyad138-B49] McLaughlin JM , Bratu DP. 2015. *Drosophila Melanogaster* Oogenesis: An Overview. In: Bratu DMG, editor. Drosophila Oogenesis. Methods in Molecular Biology, Vol. 1328. New York: Humana Press. p. 1–20. 10.1007/978-1-4939-2851-4_1 26324426

[iyad138-B50] Mével-Ninio M , Terracol R, Kafatos F. The ovo gene of Drosophila encodes a zinc finger protein required for female germ line development. EMBO J. 1991;10(8):2259–2266. doi:10.1002/j.1460-2075.1991.tb07762.x. 1712294 PMC452916

[iyad138-B51] Mito T , Nakamura T, Sarashina I, Chang CC, Ogawa S, Ohuchi H, Noji S. Dynamic expression patterns of vasa during embryogenesis in the cricket Gryllus bimaculatus. Dev Genes Evol. 2008;218(7):381–387. doi:10.1007/s00427-008-0226-z. 18542998

[iyad138-B52] Nakao H . Isolation and characterization of a Bombyx vasa-like gene. Dev Genes Evol. 1999;209(5):312–316. doi:10.1007/s004270050257. 11252184

[iyad138-B53] Ogaugwu CE , Wimmer EA. Molecular cloning and expression of nanos in the Mediterranean fruit fly, Ceratitis capitata (Diptera: tephritidae). Gene Expr Patterns. 2013;13(5–6):183–188. doi:10.1016/j.gep.2013.03.002. 23567755

[iyad138-B54] Page RE Jr , Peng CY-S. Aging and development in social insects with emphasis on the honey bee, Apis mellifera L. Exp Gerontol. 2001;36(4–6):695–711. doi:10.1016/S0531-5565(00)00236-9. 11295509

[iyad138-B55] Potts SG , Biesmeijer JC, Kremen C, Neumann P, Schweiger O, Kunin WE. Global pollinator declines: trends, impacts and drivers. Trends Ecol Evol. 2010;25(6):345–353. doi:10.1016/j.tree.2010.01.007. 20188434

[iyad138-B56] Quan H , Arsala D, Lynch JA. Transcriptomic and functional analysis of the oosome, a unique form of germ plasm in the wasp Nasonia vitripennis. BMC Biol. 2019;17(1):78. doi:10.1186/s12915-019-0696-7. 31601213 PMC6785909

[iyad138-B57] Ronquist F , Teslenko M, van der Mark P, Ayres DL, Darling A, Höhna S, Larget B, Liu L, Suchard MA, Huelsenbeck JP. Mrbayes 3.2: efficient Bayesian phylogenetic inference and model choice across a large model space. Syst Biol. 2012;61(3):539–542. doi:10.1093/sysbio/sys029. 22357727 PMC3329765

[iyad138-B58] Sagawa K , Yamagata H, Shiga Y. Exploring embryonic germ line development in the water flea, Daphnia magna, by zinc-finger-containing VASA as a marker. Gene Expr Patterns. 2005;5(5):669–678. doi:10.1016/j.modgep.2005.02.007. 15939379

[iyad138-B59] Sahai-Hernandez P , Nystul TG. A dynamic population of stromal cells contributes to the follicle stem cell niche in the *Drosophila* ovary. Development. 2013;140(22):4490–4498. doi:10.1242/dev.098558. 24131631 PMC3817939

[iyad138-B60] Sammataro D , Avitabile A. The Beekeeper's Handbook. Ithaca: Cornell University Press; 1998.

[iyad138-B61] Schindelin J , Arganda-Carreras I, Frise E, Kaynig V, Longair M, Pietzsch T, Preibisch S, Rueden C, Saalfeld S, Schmid B, et al Fiji: an open-source platform for biological-image analysis. Nat Methods. 2012;9(7):676–682. doi:10.1038/nmeth.2019. 22743772 PMC3855844

[iyad138-B62] Song X , Wong MD, Kawase E, Xi R, Ding BC, McCarthy JJ, Xie T. Bmp signals from niche cells directly repress transcription of a differentiation-promoting gene, *bag of marbles*, in germline stem cells in the *Drosophila* ovary. Development. 2004;131(6):1353–1364. doi:10.1242/dev.01026. 14973291

[iyad138-B63] Tanaka ED , Hartfelder K. The initial stages of oogenesis and their relation to differential fertility in the honey bee (Apis mellifera) castes. Arthropod Struct Dev. 2004;33(4):431–442. doi:10.1016/j.asd.2004.06.006. 18089049

[iyad138-B64] Tanaka ÉD , Hartfelder K. Sequence and expression pattern of the germ line marker vasa in honey bees and stingless bees. Genet Mol Biol. 2009;32(3):582–593. doi:10.1590/S1415-47572009005000043. 21637523 PMC3036037

[iyad138-B65] Tanaka SS , Toyooka Y, Akasu R, Katoh-Fukui Y, Nakahara Y, Suzuki R, Yokoyama M, Noce T. The mouse homolog of Drosophila vasa is required for the development of male germ cells. Genes Dev. 2000;14(7):841–853. doi:10.1101/gad.14.7.841. 10766740 PMC316497

[iyad138-B66] Tautz J . The Buzz About Bees: Biology of a Superorganism. Berlin: Springer Science & Business Media; 2008.

[iyad138-B67] Telfer WH . Development and physiology of the oöcyte-nurse cell syncytium. Adv In Insect Phys. 1975;11:223–319.

[iyad138-B68] Tsunekawa N , Naito M, Sakai Y, Nishida T, Noce T. Isolation of chicken vasa homolog gene and tracing the origin of primordial germ cells. Development. 2000;127(12):2741–2750. doi:10.1242/dev.127.12.2741. 10821771

[iyad138-B69] Van Eeckhoven J , Duncan EJ. Mating status and the evolution of eusociality: oogenesis is independent of mating status in the solitary bee Osmia bicornis. J Insect Physiol. 2020;121(Feb–Mar):104003. doi:10.1016/j.jinsphys.2019.104003 31883996

[iyad138-B70] Virtanen P , Gommers R, Oliphant TE, Haberland M, Reddy T, Cournapeau D, Burovski E, Peterson P, Weckesser W, Bright J, et al Scipy 1.0: fundamental algorithms for scientific computing in python. Nat Methods. 2020;17(3):261–272. doi:10.1038/s41592-019-0686-2. 32015543 PMC7056644

[iyad138-B71] Vreede BM , Lynch JA, Roth S, Sucena É. Co-option of a coordinate system defined by the EGFr and Dpp pathways in the evolution of a morphological novelty. EvoDevo. 2013;4(1):7. doi:10.1186/2041-9139-4-7. 23448685 PMC3621409

[iyad138-B72] Wang X , Adam JC, Montell D. Spatially localized Kuzbanian required for specific activation of Notch during border cell migration. Dev Biol. 2007;301(2):532–540. doi:10.1016/j.ydbio.2006.08.031. 17010965

[iyad138-B73] Wang SC , Ching YH, Krishnaraj P, Chen GY, Radhakrishnan AS, Lee HM, Tu WC, Lin MD. Oogenesis of hematophagous midge Forcipomyia taiwana (Diptera: ceratopogonidae) and nuage localization of vasa in germline cells. Insects. 2020;11(2):106. doi:10.3390/insects11020106. 32033475 PMC7074065

[iyad138-B74] Wang C , Dickinson LK, Lehmann R. Genetics of nanos localization in Drosophila. Dev Dyn. 1994;199(2):103–115. doi:10.1002/aja.1001990204. 7515724

[iyad138-B75] Whelan S , Goldman N. A general empirical model of protein evolution derived from multiple protein families using a maximum-likelihood approach. Mol Biol Evol. 2001;18(5):691–699. doi:10.1093/oxfordjournals.molbev.a003851. 11319253

[iyad138-B76] Xue R , Hu X, Cao G, Huang M, Xue G, Qian Y, Song Z, Gong C. Bmovo-1 regulates ovary size in the silkworm, Bombyx mori. PLoS One. 2014;9(8):e104928. doi:10.1371/journal.pone.0104928. PMC4132112 25119438

[iyad138-B77] Zhao G , Chen K, Yao Q, Wang W, Wang Y, Mu R, Chen H, Yang H, Zhou H. The nanos gene of Bombyx mori and its expression patterns in developmental embryos and larvae tissues. Gene Expr Patterns. 2008;8(4):254–260. doi:10.1016/j.gep.2007.12.006. 18267373

